# 

**Published:** 1914-11

**Authors:** 


### Some Good Things That are to Appear in the Red Back.

A paper on Pellagra, by Dr. Wilmer Allison, of Fort Worth.

Two articles on Artificial Pneumothorax, by Dr. A. G. Shortle, of Albuquerque, N. M. So far the doctor has treated 82 cases. In the first article the doctor will go into the history of the case, describe the operation, etc. In the second article he will go into details as to results, complications, etc.

Report of Four Cases of What Appeared to Be Tuberculous Meningitis, with Apparent Permanent Arrests, by Dr. Chas. C. Browning, Professor of Diseases of the Chest, University of Southern California, Los Angeles, Cal. (Nine cuts with this article.)

Stomach Troubles, by Dr. F. C. Floeckinger, of Taylor, Texas. (Five cuts with this article.)

Alcohol and the Public Health, by Dr. J. N. Hurty, State Health Commissioner of Indiana.

The Use of the Obstetrical Forceps, by T. J. Turpin, of Corpus Christi, Texas.

Editorial by Dr. George H. Lee.

An Article by Dr. L. P. Tenney, of Troup, Texas.

Editorial by Dr. Theo. Y. Hull, of San Antonio, Texas.

A paper on the work being done by the Malthusian League, by Charles V. Drysdale, Hon. Secretary, Westminster, London, England.

